# Airway Management in an Anatomically and Physiologically Difficult Airway

**DOI:** 10.7759/cureus.10638

**Published:** 2020-09-24

**Authors:** Sunny R Cai, Mani Ratnesh S Sandhu, Shaun E Gruenbaum, William H Rosenblatt, Benjamin F Gruenbaum

**Affiliations:** 1 Anesthesiology, Yale School of Medicine, New Haven, USA; 2 Laboratory Medicine, Yale School of Medicine, New Haven, USA; 3 Anesthesiology and Perioperative Medicine, Mayo Clinic, Jacksonville, USA

**Keywords:** difficult airway, airway management, pulmonary hypertension

## Abstract

A “difficult airway” should be suspected in patients with any anatomical or physiologic abnormality that might result in the loss of the airway or significant cardiopulmonary compromise upon induction of general anesthesia. Historically, an awake intubation has often been the preferred approach for airway management in these patients. Here we describe a case in which an awake intubation was safely performed in a patient with both anatomical (i.e., laryngeal mass) and physiologic (i.e., pulmonary hypertension) abnormalities. Oxygenation, airway patency, and spontaneous breathing were well maintained with successful intubation on the first attempt. We recommend that the patient’s physiologic state should always be considered in airway management planning.

## Introduction

An evidence-based approach to airway management in a patient with a “difficult airway” is paramount in clinical anesthesia practice, which has necessitated a dedicated decision pathway algorithm. Historically, a “difficult airway” describes a patient in whom the presence of anatomical characteristics may hinder adequate visualization of the vocal cords and placement of an endotracheal tube, and challenge positive-pressure mask ventilation. A “physiologically difficult airway”, albeit less described in the literature, reflects a patient with an unstable physiologic state or poor physiologic reserve that might make the patient susceptible to cardiovascular collapse and death during the period of airway securement [1]. The identification of a patient at risk for a physiological difficult airway should be considered in the preoperative evaluation and airway management planning. Particular attention should be made in determining how well hypoxia, hypotension, hypercarbia, and the anticipated time to airway securement and ventilation will be tolerated with induction of general anesthesia. Here, we report a unique case of a patient with both an anatomical (i.e., laryngeal mass) and physiological (i.e., pulmonary hypertension with impaired cardiac contractility) difficult airway. The approach to airway management is reviewed, and recommendations for similar patients with anatomical and physiologically difficult airways are discussed.

## Case presentation

An 83-year-old man presented to the Otolaryngology clinic with a one-year history of worsening dysphonia and reduced vocal projection, and worsening dyspnea over the previous seven days. Evaluation with a fiber-optic bronchoscope revealed an exophytic ulcerative mass on an immobile left vocal fold with mass extension along the anterior vocal cord commissure. The patient was scheduled to undergo direct laryngoscopy and biopsy under general anesthesia.

The patient’s past medical history was significant for a remote 20-pack-year smoking history (he quit 40 years prior), previous cerebrovascular accident (20 years prior without residual deficits), chronic atrial fibrillation on warfarin anticoagulation, non-ischemic cardiomyopathy with an ejection fraction of 35%, pulmonary hypertension, hyperlipidemia, hypertension, and secondary stage 3 chronic kidney disease. The diagnosis of pulmonary hypertension was recently made by echocardiography that demonstrated elevated right ventricular systolic pressure of 63mmHg (“moderate” in severity). The patient had never undergone a right heart catheterization. He denied a history of difficult intubation, although there were no past operative or anesthetic records available for review.

A preoperative airway assessment demonstrated a Mallampati score of II, a thyromental distance of >6 cm, several missing teeth, adequate mouth opening (>3 fingerbreadths), and unrestricted cervical spine range of motion. The risks, benefits, rationale, and approach with an awake intubation were discussed with the patient and his family, and he consented to the awake intubation.

**Figure 1:**
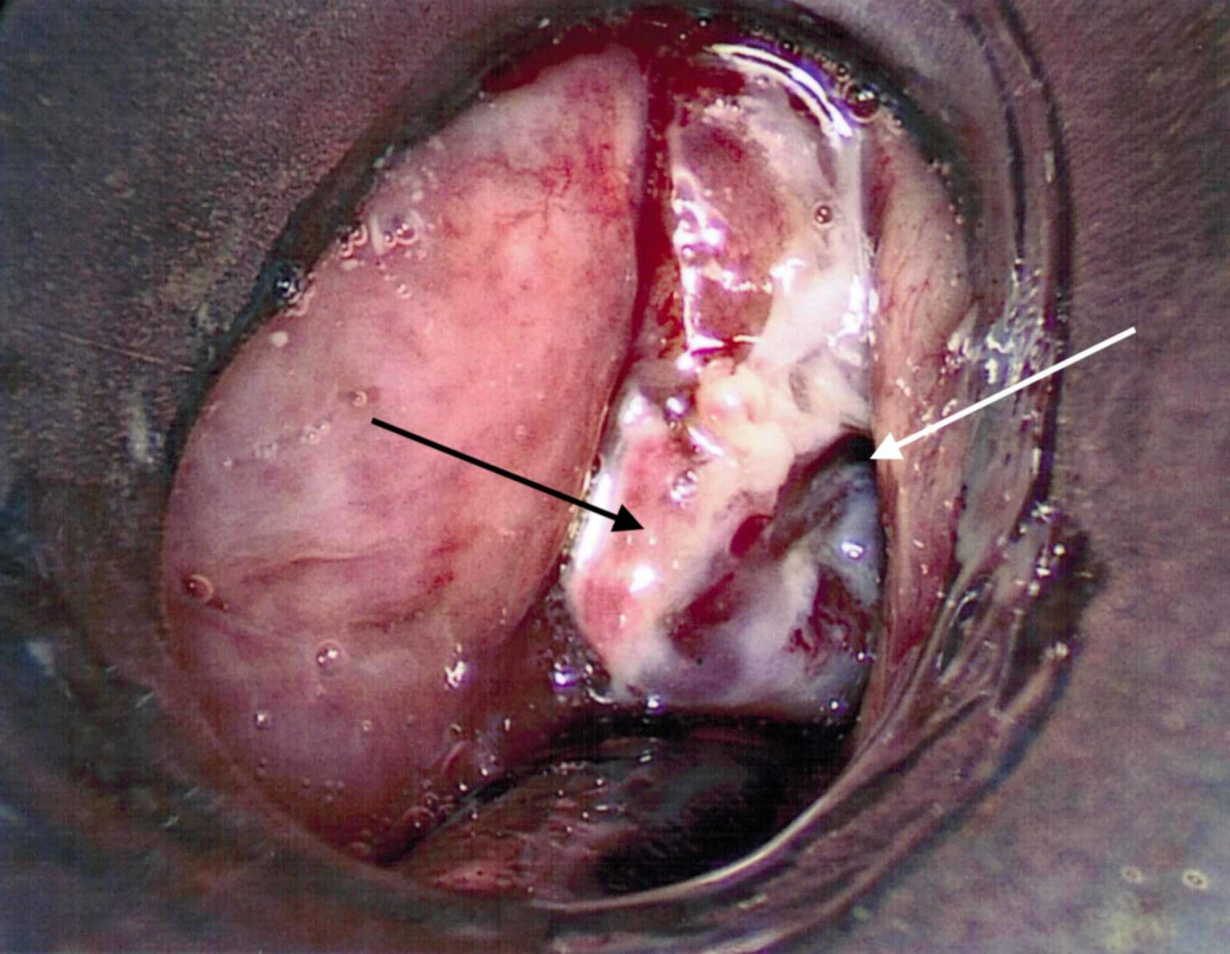
Examination of the airway mass demonstrated an ulcerative tumor on the left vocal cord with extension into the subglottic trachea. Black arrow: mass of left vocal cord; white arrow: glottic opening.

Glycopyrrolate (0.2 mg) was injected intravenously and two sprays of oxymetazoline were administered in each nostril. The patient was asked to extend his tongue which was held in extension with gauze. Topicalization of local anesthetic to the oropharynx was applied with 8 mL viscous 2% lidocaine, via 10cc syringe and 14 gauge plastic angiocatheter, followed by 10 mL of 4% lidocaine sprayed onto the oral mucosa with an atomizer tip. The patient was encouraged to “take deep breaths” to facilitate entrainment of local anesthetic to and below the vocal cords. In the operating room, an intravenous dexmedetomidine infusion (1 mcg/kg/hr) was initiated and the patient was positioned with the head of the bed elevated. The patient remained spontaneously ventilating, conversant, and able to follow commands. Tracheal intubation was performed with a 6.5-mm cuffed endotracheal tube (ETT) over a flexible bronchoscope and the patient tolerated the intubation without physiologic decompensation. Sevoflurane was used for induction and maintenance of general anesthesia. An examination of the airway mass demonstrated an ulcerative tumor on the left vocal cord with extension into the subglottic trachea (Figure 1). A biopsy and surgical debulking of the tumor was performed. At the end of the procedure the patient was fully awake, following commands, and was extubated uneventfully. The patient demonstrated adequate ventilation in the postoperative period without supplemental oxygen, and was able to swallow without difficulty. He was discharged to home on the same day. 

## Discussion

The “physiologically difficult airway”, first described by Mosier and colleagues in 2015 [1], characterized by four physiologic derangements that can predispose a patient (with or without anatomical abnormality) to cardiovascular collapse and even death during airway management: hypoxemia, hypotension, severe metabolic acidosis, and right ventricular failure. In a recent multicenter study, nearly 30% of critically ill patients demonstrated cardiovascular collapse after induction of general anesthesia and intubation [2]. Therefore, anesthesiologists should be aware of the characteristics that define the physiologically difficult airway and be able to identify any potential risk factors. Here, we presented the considerations and management of a unique patient that presented both an anatomical and physiologically difficult airway.

Our patient was considered a physiologically difficult airway because of his history of pulmonary hypertension. Our intraoperative management plan focused on maintaining right ventricular cardiac output, avoiding systemic hypotension, and avoiding all circumstances that could contribute to exacerbating pulmonary hypertension (hypoxemia, hypercapnia, acidosis, hypothermia, and hypervolemia). Preoperatively, we considered how induction of general anesthesia, apnea, and intubation might not be well tolerated considering his poor physiologic state. In the presence of acute hypoxia, hypoxic pulmonary vasoconstriction would likely worsen his pulmonary hypertension and result in acute right heart failure [3]. With impaired cardiac contractility at baseline, an acute worsening of his right ventricular function might have been catastrophic and result in cardiovascular collapse. We had vasodilators, inotropes, and vasopressors readily available to maintain right ventricular function and reduce right ventricular afterload but ultimately, we did not have to utilize any vasoactive agents since our patient remained hemodynamically stable. Fluid management was carried out in a restrictive and targeted manner to optimize right-ventricular preload.

Induction and maintenance of general anesthesia with sevoflurane was likely the safest approach in this patient for several reasons. First, compared with propofol, volatile anesthetic induction would likely provide favorable hemodynamics in this patient with baseline right ventricular dysfunction [4]. Second, sevoflurane induction would facilitate the maintenance of spontaneous breathing during the procedure, which is important because right ventricular function could further worsen in the setting of increased intrathoracic pressure that accompanies positive pressure ventilation. In this patient, awake intubation was preferred because of the presence of an anatomically abnormal airway, which presented a risk of airway obstruction during a routine induction of general anesthesia.

Another benefit of an awake intubation in this patient was its likely first-pass success. The incidence of adverse events leading to hypoxemia and hemodynamic collapse with intubation is higher in patients with underlying physiologic derangements, and first-pass success is critical to avoid further decompensation [5]. This patient was also unlikely to tolerate multiple intubation attempts due to the ulcerative lesion on his left vocal cord. With multiple intubation attempts, mechanical trauma to the lesion could result in a high risk of bleeding, thereby obscuring the view and further threatening the airway. Additionally, with a laryngeal mass we anticipated the high likelihood of a difficult mask-ventilation, which further necessitated an induction plan that would preserve the respiratory drive. Therefore, for both physiologic and anatomic reasons, first-pass success in this patient was crucial.

## Conclusions

In every patient, the airway management plan should include considerations of both the patient’s airway anatomy as well as the identification of derangements that might suggest a physiologically difficult airway. Here we described our approach and rationale for airway management in a complicated patient with both an anatomically and physiologically difficult airway. For a patient with similar physiologic and anatomic presentation, we recommend induction and intubation methods that maintain spontaneous ventilation, avoid hemodynamic instability, and aim for first-pass intubation. Future studies are required to define the incidence and the management of the physiologically difficult airway in different settings to prevent catastrophic, and potentially fatal complications.
